# *Yersinia pestis* strains from Latvia show depletion of the *pla* virulence gene at the end of the second plague pandemic

**DOI:** 10.1038/s41598-020-71530-9

**Published:** 2020-09-03

**Authors:** Julian Susat, Joanna H. Bonczarowska, Elīna Pētersone-Gordina, Alexander Immel, Almut Nebel, Guntis Gerhards, Ben Krause-Kyora

**Affiliations:** 1 Institute of Clinical Molecular Biology, Kiel University, Rosalind-Franklin-Straße 12, 24105 Kiel, Germany; 2 Institute of Latvian History, University of Latvia, Kalpaka bulvāris 4, Riga, 1050 Latvia

**Keywords:** Evolution, Diseases, Pathogenesis

## Abstract

Ancient genomic studies have identified *Yersinia pestis* (*Y. pestis*) as the causative agent of the second plague pandemic (fourteenth–eighteenth century) that started with the Black Death (1,347–1,353). Most of the *Y. pestis* strains investigated from this pandemic have been isolated from western Europe, and not much is known about the diversity and microevolution of this bacterium in eastern European countries. In this study, we investigated human remains excavated from two cemeteries in Riga (Latvia). Historical evidence suggests that the burials were a consequence of plague outbreaks during the seventeenth century. DNA was extracted from teeth of 16 individuals and subjected to shotgun sequencing. Analysis of the metagenomic data revealed the presence of *Y. pestis* sequences in four remains, confirming that the buried individuals were victims of plague. In two samples, *Y. pestis* DNA coverage was sufficient for genome reconstruction. Subsequent phylogenetic analysis showed that the Riga strains fell within the diversity of the already known post-Black Death genomes. Interestingly, the two Latvian isolates did not cluster together. Moreover, we detected a drop in coverage of the pPCP1 plasmid region containing the *pla* gene. Further analysis indicated the presence of two pPCP1 plasmids, one with and one without the *pla* gene region, and only one bacterial chromosome, indicating that the same bacterium carried two distinct pPCP1 plasmids. In addition, we found the same pattern in the majority of previously published post-Black Death strains, but not in the Black Death strains. The *pla* gene is an important virulence factor for the infection of and transmission in humans. Thus, the spread of *pla*-depleted strains may, among other causes, have contributed to the disappearance of the second plague pandemic in eighteenth century Europe.

## Introduction

*Yersinia pestis* (*Y. pestis*) is the causative agent of plague that evolved from a relatively benign pathogen—*Yersinia pseudotuberculosis*—thousands of years ago^1–4^. Whilst plague is a zoonotic disease with rodents being the primary hosts of the pathogen^5–7^, it has also afflicted humans for at least 5,000 years^1^. In the last two millennia, *Y. pestis* was responsible for three major epidemics, of which the second was the most infamous. This pandemic began with the Black Death in the fourteenth century (1,346–1,353). It is thought to have originated in Asia from where it quickly spread to and across the European continent killing approximately 30–50% of the population within a few years^8,9^. The second pandemic persisted for over 400 years^10^ causing, among others, the Great Plague of London (1,665–1,666) and Marseille (1,720–1,722)^11^. In contrast to western Europe, the first major outbreaks of plague in the north-eastern part of the continent took place mainly in the post-Black Death period (after 1,353), for instance in Riga (1,601–3, 1,621–23 and 1,657) or during the Russian Plague (1,770–1,772)^12–14^.

Ancient DNA (aDNA) studies have shown that all second-plague pandemic strains cluster together in branch 1 of the *Y. pestis* phylogeny, close to present-day representatives^15–17^. Interestingly, pathogens involved in the post-Black Death outbreaks derived from two Black Death strains from London and Barcelona^15,18^. This post-Black Death subset includes strains from Germany (Starnberg 1,433–1,523, Ellwangen 1,486–1,627), England (Cambridge 1,433–1,523, London 1,560–1,635), Sweden (Stans 1,485–1,635) and France (Marseille 1,720–1,722)^19^. Since no modern descendants have yet been identified, this lineage probably went extinct^10^.

There are ongoing debates about the reasons as to why the disease disappeared from Europe in the eighteenth century. Improvements in living conditions, hygiene, and quarantine facilities as well as a replacement of the rodent host from black to brown rat have been suggested^20^. Other explanations revolved around the idea that evolutionary processes may have favored less virulent pathogen strains^21^ or may have resulted in increased immunity of the human population^20^.

Most of the second-pandemic strains analyzed to date have been isolated from human remains excavated in western Europe, with exception of two strains found in Russia (Bolgar 1,362–1,400^18^, Laishevo 1,300–1,400^19^). Therefore, not much is known about the diversity and microevolution of the strains responsible for disease outbreaks in eastern Europe, which could have served as a gateway for the spread of the pathogen towards the west.

**Figure 1:**
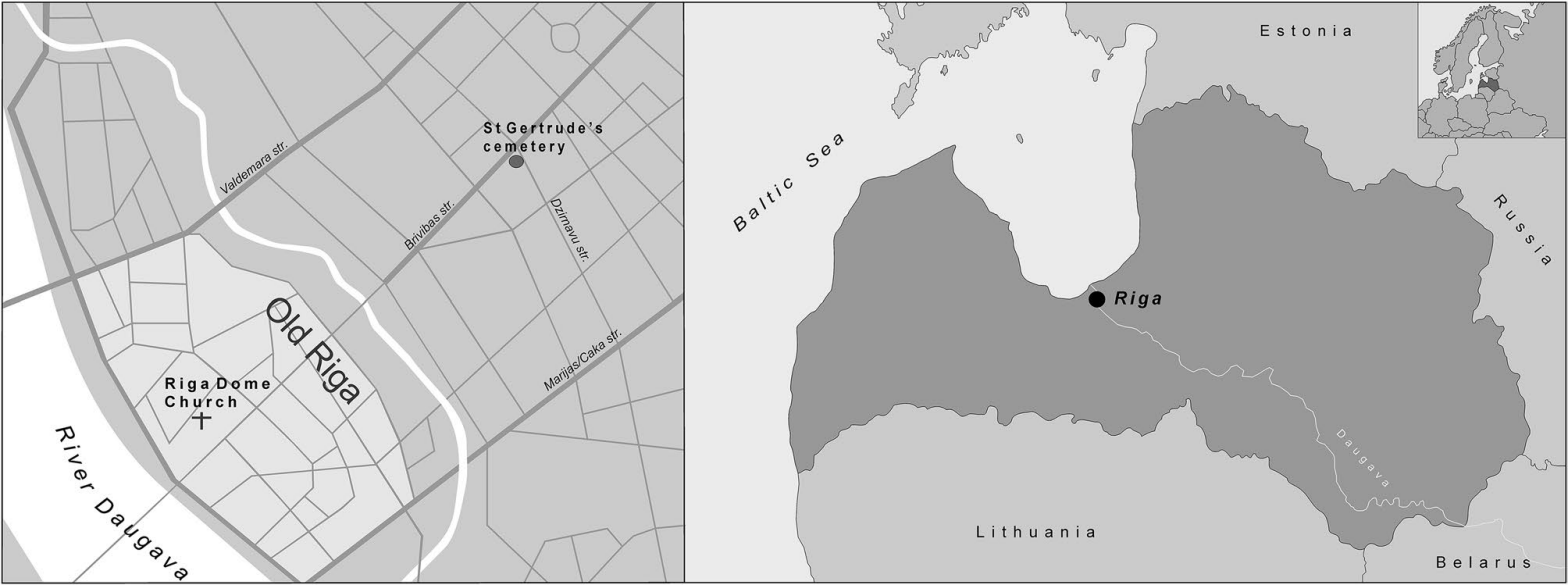
Cemeteries in Riga from which the samples in this study were obtained (left picture). Map of Latvia and Europe (right picture, upper right corner) with the location of Riga. (Figure was created using CorelDRAW Home & Student X8, URL: https://www.coreldraw.com/en/).

At least three plague outbreaks in Riga (Latvia) dating to the seventeenth century have been previously reported^12,13^. To confirm the presence of *Y. pestis* in Riga, we analysed samples from 16 human skeletons excavated from two cemeteries (fifteenth–seventeenth century^13^) (Fig. 1 and Table 1). The individuals had been interred in multiple or mass burials and single graves (Table 1). Four of the samples yielded positive screening results for the presence of *Y. pestis*, leading to the successful reconstruction of two complete bacterial genomes. This new data allowed us to expand the geographical range of known *Y. pestis* strains to north-eastern Europe.

**Table 1:** List of samples from St. Gertrude’s Church cemetery and Riga Dome Church cemetery.

Cemetery	Context	Burial no.	Osteological sex	Genetic sex	Age (years)	*Yersinia pestis*
St. Gertrude’s Church cemetery	Mass grave 1	G83	ND	No DNA	11–13	No DNA
		G157	M	No DNA	30–35	No DNA
		G488	M	M	30–35	+
		G645	F	F	20–25	+
	Mass grave 2	G103	ND	M	10–11	+
		G177	ND	M	10–12	−
		G687	M	M	45–50	−
	Burial pit	G691	ND	F	9–11	−
		G701	F	F	35–40	+
	General cemetery	G41	ND	M	14–15	−
		G92	M	M	55–60	−
		G100	M	M	20–25	−
Riga Dome Church cemetery	Mass burial pit	G143b	F	F	25–30	−
		G161c	F	M	50–60	−
		G171c	F	ND	40–50	−
	Collective burial pit	G184b	M	M	40–50	−

*M* male, *F* female, *ND* not determinable; + /*− Y. pestis-*positive or negative.

## Results

Sixteen individuals excavated from two cemeteries located in Riga, Latvia were submitted for aDNA analysis. For 14 samples, authentic ancient shotgun sequences were generated. Of these, we identified four *Y. pestis*-positive individuals using MALT in BlastN mode (Table 1 and S1). Three of them (G103, G645, G488) were buried in two mass graves and one (G701) in the burial pit of the St. Gertrude’s Church cemetery. The presence of *Y. pestis*-specific reads was confirmed by mapping against a multi fasta reference containing several representatives of the genus *Yersinia* (Table S2). Furthermore, we calculated an endogenous based score (i.e. the number of *Y. pestis*-specific reads with respect to the number of total reads in the sample)^2^ (Table S3). Two of the samples (G488, G701) were identified as strong positives (endogenous based score > 0.005, Table S3). For these candidates, we subsequently generated 330 and 383 million reads, respectively, to reconstruct the complete bacterial genomes. The obtained reads were mapped against the *Y. pestis* reference (CO92, NC_003143.1) containing the chromosome and all three plasmids (pCD1, NC_003131.1; pMT1, NC_003134.1; pPCP1, NC_003132.1). Both samples exhibited a genome coverage above 92% for the chromosome as well as for the three plasmids (Table 2 and Fig. S1). Terminal deamination patterns displayed damage profiles typical of authentic aDNA (Fig. S2).

**Table 2:** Basic mapping statistics for the strongly positive *Y. pestis* samples after duplicate removal and quality filtering.

Sample	Aligned reads	Coverage ≥ 1x (%)	Coverage ≥ 2x (%)	Coverage ≥ 3x (%)	Mean coverage whole genome
**Chromosome**					
G701	237,557	92.74	75.66	53.77	3.09x
G488	597,757	98.85	95.83	89.76	6.79x
**pMT1**					
G701	6,540	97.74	89.33	75.03	4.16x
G488	17,450	99.65	99.04	97.52	9.68x
**pPCP1**					
G701	4,544	98.75	94.42	88.24	30.07x
G488	11,893	99.80	99.14	98.72	68.28x
**pCD1**					
G701	6,659	99.35	96.30	89.55	5.9x
G488	26,728	99.99	99.14	98.72	20.99x

Mapping against the CO92 chromosome (NC_003143.1), pMT1 (NC_003134.1), pPCP1 (NC_003132.1) and pCD1 (NC_003131.1).

**Figure 2:**
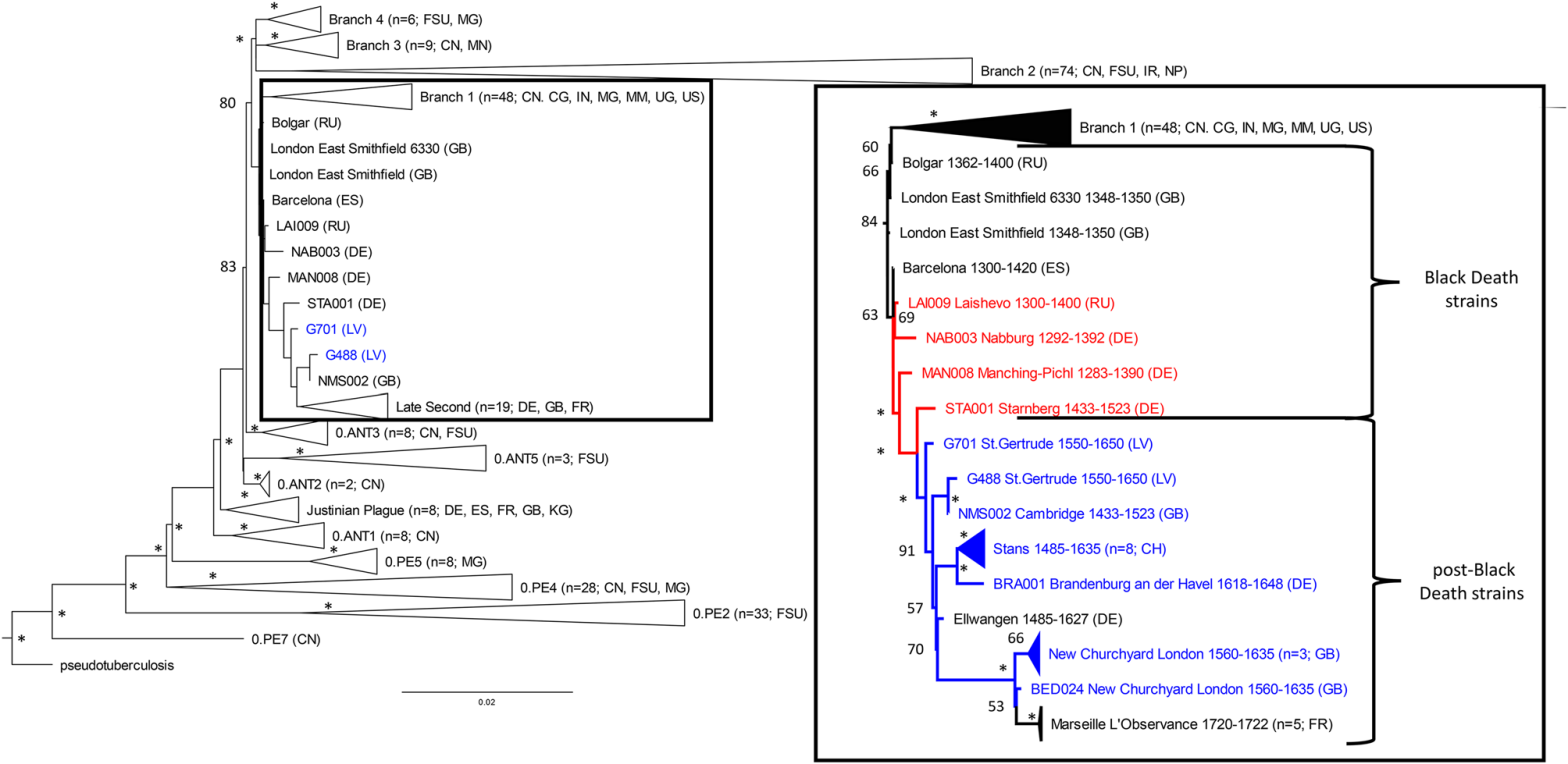
Maximum-likelihood tree. The tree is based on the SNP alignment (15,976 positions) of 228 modern *Y. pestis* genomes, 36 published ancient *Y. pestis* strains, one *Y. pseudotuberculosis* genome, and the two genomes G488 and G701 from St. Gertrude’s Church cemetery (left panel, blue). Country abbreviation is given in brackets (DE = Germany, ES = Spain, FR = France, GB = Great Britain, US = United States, RU = Russia, LV = Latvia, CN = China, CG = Congo, FSU = Former Soviet Union, IN = India, IR = Iran, MG = Madagascar, MM = Myanmar, MN = Mongolia, NP = Nepal, UG = Uganda, KG = Kyrgyzstan, CH = Switzerland). Bootstrap values are shown on the nodes for 500 replicates. An asterisk (*) indicates a bootstrap support above 94. Strains in red exhibit no signs of the *pla-*negative plasmid. Strains in blue exhibit clear signs of the *pla-*negative plasmid. For the strains in black (within the Back Death and post-Black Death subclades), the *pla* status was inconclusive or could not be determined due to a general lack of coverage.

We computed maximum likelihood and Bayesian trees (Figs. 2, S3 and S4) with the two genomes from Riga (G488, G701) and previously published *Y. pestis* strains (228 modern (Table S4), 36 ancient (Table S5)) and a Y. *pseudotuberculosis* genome (NZ_CP008943.1). The strains from Riga clustered with other second-plague pandemic strains from Starnberg (STA001) and Cambridge (NMS002)^19^ with a strong bootstrap support of > 97% and a posterior probability of 1 (Figs. 2, S3 and S4).

Both Riga isolates had SNP alleles (p1 and p2^14^) diagnostic for branch 1 (Table S6). Furthermore, they showed differences in the SNPs p3 and p4 relative to the CO92 reference sequence. These substitutions also characterize other previously reported second-plague pandemic strains from London (England), Barcelona (Spain), Ellwangen (Germany), and l’Observance (France)^10,15,18^.

**Figure 3:**
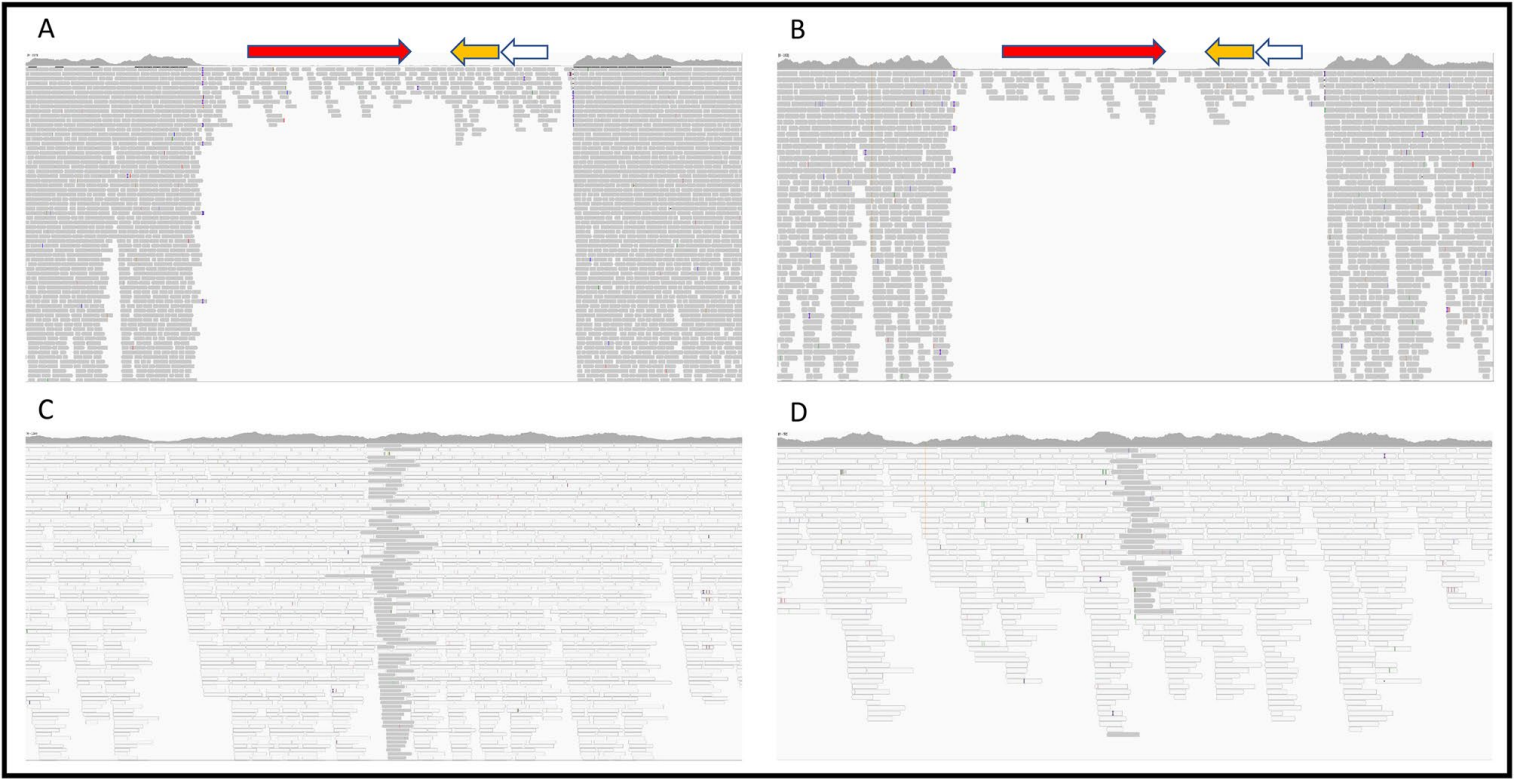
Coverage plots of pPCP1. (**A**, **B**) Coverage plot of the *pla* region in G701 (**A**) and G488 (**B**) depicting the *pla* gene in red, the putative transcriptional regulator in yellow and the hypothetical protein in white. G488 average coverage (ac) before the *pla* region 107x, ac of the *pla* region 7x; G701 ac before the *pla* region 47x, ac of the *pla* region 3x. Each grey line represents an obtained aDNA sequence. (**C**) Coverage plot showing reads of G701 (sequences in dark grey) specifically mapping to the gap-spanning region. A white color indicates sequences that could be mapped to both the *pla*+ and *pla− *references as they are not specific to the newly created region. (**D**) Coverage plot showing reads of G488 (sequences in dark grey) specifically mapping to the gap-spanning region.

The two reconstructed genomes from Riga exhibited a drastic drop in coverage of sequences mapping to the *pla* gene region (NC_003132.1:6,428–8,530), which consists of the *pla* gene, a putative transcriptional regulator and a hypothetical protein on the pPCP1 plasmid (Fig. 3). We confirmed the presence of two variants of the plasmid, i.e. one with (*pla*+) and one without (*pla*−) the *pla* region, by identifying gap-spanning reads in each of the strains (Figs. 3C,D, S5). In contrast, we noted only one bacterial chromosome per strain, indicating that the two distinct pPCP1 plasmids existed in the same bacterium. To rule out that the *pla* gene originated from contamination, a competitive mapping against the *pla* gene from environmental bacteria (e.g. *E. coli* and *Citrobacter koseri*) and *Y. pestis* showed that the reads in this region were indeed specific for *Y. pestis* and exhibited the expected damage patterns (Table S7). This observation was verified by a competitive mapping against a *pla*+ and a *pla− *reference (Fig. 3 and Table S8). The observed ratio between the coverage of the region in *pla*+ *and pla− *plasmids was about 1:10 (Table S9). To further explore this finding, we re-analyzed 28 previously published *Y. pestis* genomes from the second pandemic^10,15,18,19^. Interestingly, in 14 strains (marked in blue, Fig. 2) we detected gap-spanning reads and a depletion of the *pla* region, also indicating the presence of both *pla*+ and *pla− *plasmids (Fig. S6 and Table S10). Four more strains showed no signs of *pla− *plasmids (marked in red, Figs. 2 and S6), whereas ten others, for which the overall pPCP1 plasmid coverage was poor, were excluded as their *pla* region could not be examined reliably (marked in black, Fig. 2). The possibility that the different plasmids could be the result of a co-infection with two different strains is highly unlikely as this pattern was found in several *Y. pestis* genomes from various sites and times, and there was no evidence for such an infection on the chromosomal level.

With regard to (potentially) functional SNPs, we identified two variants of interest. First, both Riga genomes carried the T259 allele in the *pla* gene that is important for massive bacterial dissemination in the bubonic form of plague^22^. Second, in sample G488, SnpEff^23^ detected a variant in the s*srA-smpB* region.

For five of the samples (G92, G143b, G161c, G488 and G701), the human endogenous DNA content in the metagenomic data was sufficient to investigate the population genetic ancestry using PCA, f3 and admixture analyses. The results indicate a northern European origin of the individuals who were genetically fairly homogeneous (Figs. S7, S8, S9 and S10).

## Discussion

Here we analyzed the remains of 16 individuals who had been interred in two cemeteries (Dome Church cemetery, St. Gertrude’s Church cemetery) in Riga, Latvia (fifteenth–seventeenth century) (Fig. 1 and Table 1). Four of the individuals buried in two mass graves and a burial pit of St. Gertrude’s Church cemetery were infected with the *Y. pestis* bacterium. Previous analyses led to the hypothesis that people inhumed in the mass graves were rural immigrants. Our human population genetic data confirmed their northern European descent. Most likely they came to Riga during the famine of 1601–2 and stayed in shelters in the vicinity of St. Gertrude’s Church^13^. Inadequate nutrition and crowded, unhygienic conditions may have increased their susceptibility to infectious diseases, including plague^24,25^. Our finding showed that the individuals buried in the mass graves as well as the small burial pit were indeed victims of plague^26,27^. Thus, we corroborate historical reports of local plague outbreaks in Riga at the beginning of the seventeenth century (1,601–3, 1,621–23 and 1,657)^12^.

From two (G488 and G701) of the four infected individuals, high-quality whole genomes of *Y. pestis* were generated. They formed a subclade in branch 1 with other strains dating to the post-Black Death pandemic (Fig. 2). They all originated from Black Death genomes from London^15^ and Barcelona^18^. Although the two Riga strains were found in the same cemetery (in one mass grave and the burial pit), they showed a surprisingly large genetic distance. Based on this observation and the close proximity of G488 to NMS002 (Cambridge^19^) (Fig. 2), we assume that G488 had not evolved from G701 during a single outbreak. This may indicate that the two different strains were responsible for two subsequent plague events^12,13^.

As no modern strains fall into the post-Black Death subclade, which includes the two genomes from Riga, it could represent a *Y. pestis* lineage that went extinct^10^. Although the strains differ widely in terms of time and geography, their positions within the subclade—like pearls on a string—suggest continuous in situ evolution. This observation agrees with the hypothesis that the second plague pandemic reached Europe from Asia rather in one than in multiple waves. If the pandemic had multiple sources, one would expect a higher diversity. Thus, the post-Black Death outbreaks were likely due to established plague reservoirs within Europe. Little is known about natural plague foci and hosts during the second pandemic. Several rodent species, e.g. marmots and squirrels, from the Alps and the steppe have been mentioned as possible reservoirs^20^.

The majority (ratio 10:1) of the pPCP1 plasmid copies carried by the two Riga strains G488 and G701 did not possess the *pla* region. Based on the chromosome data we did not see any signs of coinfection with different strains that could explain the presence of two different pPCP1 plasmids. In a re-evaluation of already published second-plague pandemic genomes, we identified 14 strains carrying both plasmid types (*pla− *and *pla*+) (Table S10, Fig. S6). All 14 strains were dated to the post-Black Death period whereas four strains which only had copies of the *pla*+ plasmid were present during the Black Death (Fig. 2). The *pla* region consists of the *pla* gene, a putative transcriptional regulator and a hypothetical protein. Up to now, the role of the putative transcriptional regulator and the hypothetical protein with respect to *pla* expression and function has remained unclear. Several studies have shown that *pla* is an important factor for the infection of and transmission in humans. The pla protease activates host fibrinolysis and allows the spread of the bacteria to the lymphatic nodes^28^ as well as their extensive growth in the lower airways^29,30^. *Pla−* mutants of *Y. pestis* have been associated with reduced dissemination and virulence^22,30,31^. In mice, *pla− *strains transmitted by fleas were reported to cause primary septicemic plague while no bubonic forms were detected^31^. Therefore, *pla* seems to influence the infection routes leading to bubonic and pneumonic plague, while the septicemic route remains unaffected^32^. Therefore, the extremely high mortality of the Black Death could possibly be explained by the functional features of *pla*. The pandemic was mainly associated with a rapid spread of the severe bubonic form via the flea vector. In contrast, subsequent outbreaks could have been influenced by a slower transmission rate and a different disease course (primarily septicemic rather than bubonic) due to the *pla-*depleted strains (Fig. 2).

Horizontal gene transfer, selective gene loss, and changes in regulatory pathways have been described in the evolution and adaptation of *Y. pestis* to new host environments^33,34^. These mechanisms together with the genomic structure (e.g. IS-like elements or transposases) of the plasmids could have led to a loss of *pla* or a *pla-*depletion. *Pla−* *Y. pestis* strains have been isolated worldwide^35,36^ and *pla−* plasmids are known from *Pestoides* strains^37^—enzoonotic pathogens that infect rodents^38^, but rarely humans^39^. The *pla*-depleted post-Black Death strains could therefore be an adaptation to a specific host environment, perhaps in rodents.

The strain from individual G488 possessed a mutation in the s*srA* gene, which has not yet been described in other strains. Together with the smpB protein, ssrA creates a complex responsible for maintaining cellular homeostasis^40–42^. The important role of this system in bacterial pathogenicity was shown in previous studies on several bacteria such as *Salmonella*, *Y. pseudotuberculosis* and *Y. pestis*^43–45^. Interestingly, in a mouse model, *Y. pestis* strains with a deletion of s*srA* and *smpB* had limited ability to disseminate and colonize target organs. Moreover, mice which were intranasally immunized with this mutated strain were protected against pulmonary infection with a fully virulent strain^46^. In view of these findings, a SNP in the *ssrA* gene is intriguing. However, the functional implications of this single point mutation remain unclear.

*Y. pestis* strains responsible for the Post-Black Death plague showed a *pla*-depletion in the pPCP1 plasmid. This change in the pathogen genome can probably be attributed to an adaptation of the bacterium to a new host environment (e.g. rodents). In humans, the depletion may have favorably influenced the disease course, the probability of disease manifestation after a flea bite and/or the transmission rate. These positive effects fit with the evolutionary hypothesis that less virulent strains may, at least in part, explain the easing and ultimate disappearance of plague from Europe^21^. Nevertheless, it is imperative to obtain more *Y. pestis* genomes and conduct additional functional studies to create a more comprehensive picture of the bacteria responsible for the second plague pandemic.

## Material and methods

### Historical background and sample selection

Individuals excavated from the cemeteries Riga Dome Church and St. Gertrude’s Church cemetery, Riga, were selected for a bacterial pathogen screening (Fig. 1). At Riga Dome Church cemetery, three mass burial pits were found at the northern wall of the church. A total of 87 individuals were identified and archaeological finds suggest that they date to the seventeenth century^47^. Unlike people buried elsewhere in the cemetery, the individuals in the mass burial pits had been buried in coffins, and none of them showed evidence of peri-mortem trauma^48^. Therefore, it is likely that they had succumbed to a mass mortality event that was not related to violence. Indeed, they could be victims of one of the seventeenth century plague epidemics. Historical sources suggest that during the plague epidemic of 1,657, the churches of Riga ran out of intramural burial space, which was normally reserved for people of a higher social status^48^*.* It is possible that during the epidemic, the dead were buried in larger pits outside the church, albeit in a dedicated area and in coffins to signify their higher social status. In addition, smaller collective burials close to the aforementioned mass burial pits were found. In these, four to nine people were buried in several layers, without coffins. For DNA analysis, three samples were selected from the mass burial pits with coffins, and one from the smaller collective burial pits (Table 1).

The other site selected for aDNA analysis was St. Gertrude’s Church cemetery. The graveyard was in use between the fifteenth and seventeenth century, and back then located outside the city wall (Fig. 1). The cemetery was divided into four distinct burial areas: a general cemetery, two mass graves, and a small mass burial pit. In total, 709 individuals were excavated from this cemetery, and 283 of these were buried in the two mass graves (south-eastern mass grave, or mass grave 1, and north-western mass grave, or mass grave 2)^13^. The bodies in the mass graves were placed on top of each other, reaching seven to eight layers in total. Both archaeological (based on artefacts) and radiocarbon analysis dated the two mass graves to the end of the sixteenth or the beginning of the seventeenth century, although they are not necessarily contemporaneous^49^. The chronology of the small mass burial pit with 15 buried individuals is not entirely clear. The demographic profile of the mass graves with comparatively few children < 7 years of age and, again, the lack of evidence of peri-mortem trauma, suggested that these people had died in a non-violent mass mortality event^13,48^. Moreover, the date of both mass graves coincides with two historically documented plague epidemics (1,601–3 and 1,621–23) and a famine (1,601–2)^12^. For DNA analysis, four samples were selected from mass grave 1, three from mass grave 2, two from the grave pit and three from the general cemetery (Table 1).

### DNA isolation, sequencing and postprocessing

All lab work was carried out in a dedicated ancient DNA facility. DNA extraction of 16 tooth samples from Riga, sequencing (Illumina HiSeq4000 (2 × 75 bp)) and subsequent clip and merge was performed as described in Krause-Kyora et al.^50^. Reads that were shorter than 25 bp were discarded after merging. Two samples (G83 and G157) did not yield sufficient DNA and were not processed further (Table 1).

### Pathogen screening

Screening of the 14 samples was carried out with the software MALT using a custom database consisting of complete bacterial genomes downloaded from NCBI (24.01.2019)^51^. A sequence identity threshold of 90% was set and the alignment mode was changed to SemiGlobal. The analysis was done using the following command:

malt-run -mode BlastN -e 0.001 -id 85 -alignmentType SemiGlobal -index $index -inFile $FASTQCM -output $OUT

where $index is the index file, $FASTQCM is the clipped and merged file and $OUT is the output file.

The resulting alignments were visually inspected using MEGAN 6^52^.

### *Y. pestis* alignment

All four positive samples from the screening (G103, G488, G645, G701) were mapped against the *Y. pestis* reference genome including the three plasmids pCD1, pMT1 and pPCP1 (NC_0031431.1; NC_003131.1; NC_003134.1; NC_003132.1). The mapping was conducted using BWA with the following command line^53^:

bwa aln -n 0.01 -l 300 $INDEX $FASTQCM $OUT

where $INDEX is the reference, $FASTQCM is the input file and $OUT is the output file. Minimum mapping quality was set to 0. Subsequent duplicate removal was performed using DeDup version 0.11.3, part of the EAGER pipeline^54^, with default parameters. Subsequently, the Binary Alignment Map (BAM) files were filtered for a mapping quality of 30 using the following command in samtools^55^:

samtools view -bq $INT $IN > $OUT

where $INT is the mapping quality, $IN is the input BAM file and $OUT is the output BAM file.

### Sample authentication

To further authenticate the positive samples, an endogenous score was used to calculate the number of *Y. pestis*-specific reads with respect to the number of total reads in the sample^2^. Positive samples were mapped against a multi fasta reference containing several representatives of the genus *Yersinia* (Table S2) and the three *Y. pestis-*specific plasmids (pCD1, pMT1, pPCP1). The mapping was done as described above followed by a duplicate removal step. Mapped reads were filtered for a mapping quality greater than 30 using samtools as described above.

Applying samtools idxstats, an endogenous based score was used to evaluate the potential of the sample being positive for *Y. pestis*. The score was calculated with the following formula:

$$\frac{\left(YPS-\left(YS\right)\right)}{M}\times 1000$$

where YPS is the number of reads specifically mapping to *Y. pestis*; YS is the maximum number of reads mapping specifically to any *Yersinia* species with the exception of *Y. pestis* and M is the total number of merged reads in the sample. By using the maximum number of reads mapping to another species of the genus *Yersinia*, the score takes into account different sources of contamination other than *Y. pseudotuberculosis*. Two of the four positive samples exhibited a score higher than 0.005, a value which indicates a strong positive candidate (Table S3).

### VCF-generation and phylogenetic analysis

VFC files were generated for the two positive samples using GATK and the *Y. pestis* reference with the following command line^56^:

GenomeAnalysisTK.jar-T UnifiedGenotyper -R $REF -I $IN -out_mode EMIT_ALL_SITES -o $OUT

where $REF is the reference, $IN is the input BAM file and $OUT is the VCF output. These two VCFs together with a VCF dataset of 264 strains (Table S4 and S5, mapped (see *Y. pestis* alignment) and generated as described above) and one *Y. pseudotuberculosis* (NZ_CP008943.1) genome were used as an input for the MultiVCFAnalyzer to generate a SNP-based multiple alignment^57^. The analysis was carried out with the following command:

Java -jar MultiVCFAnalyzer_0-85-1.jar NA $REF NA $OUT F 30 3 0.9 0.9 NA

where $REF is the *Y. pestis* reference and $OUT is the output folder. *Y. pseudotuberculosis-*specific variants were removed from the SNP table to improve visual resolution of the tree. The resulting SNP table was also filtered for SNPs in repetitive regions and homoplasies^16^. The SNP table was reformatted to multi fasta and nexus format that were used as input for RAxML and MrBayes^58,59^. RAxML was executed with the following command line:

RaxmlHPC -f a -x 12345 -p 12345 -#500 -m GTRGAMMA -s $IN -o $OG -n $NAME

where $IN is the input file, $OG is the name of the outgroup and $NAME is the outfile name.

MrBayes was executed applying the GTR model with the default number of generations.

Resulting trees were visualized using FigTree^60^.

### SNP effect analysis

VCFs from the Y. *pestis-*positive samples were filtered for SNPs with a coverage of at least 3x, a calling quality of 30 and a 90% majority call. This set of filtered SNPS was provided to SNPEff to analyze the effects of SNPs in the ancient genomes^23^. SNPEff was executed with the following command line:

java -jar snpEff.jar $REF $IN > $OUT

where $REF is the SNPEff reference, $IN is the input VCF file and $OUT is the annotated SNPEff output file. The annotated VCF file was filtered for high-impact SNPs and evaluated.

### pPCP1 and *pla* analysis

To ascertain that the coverage in the low-coverage region was not introduced artificially by environmental contamination, we carried out a competitive mapping as described above. The fasta reference that was used contained the *Y. pestis* reference sequence of the pPCP1 plasmid and also the *pla* genes of *Citrobacter* and *E. coli* (Table S7). Gap-bridging reads were analyzed by removing the low-coverage region from the pPCP1 plasmid. Seqkit was used with the following command lines^61^:

seqkit subseq -r 1:6428 $IN > $OUT1

seqkit subseq -r 8530:9612 $IN > $OUT2

where $IN is the pPCP1 reference (NC_003132.1) and $OUT1/$OUT2 are parts of the new plasmid without the *pla* region. Both output files were concatenated to form a complete plasmid. An additional mapping with the pPCP1 plasmid from which the *pla* region was removed (*pla-)* and the regular pPCP1 plasmid (*pla*+) as references was performed (Table S8). These references were used to identify reads which were truly unique for the new region in the *pla− *plasmid. All the non-specific reads received a mapping quality of zero as the exact same region was present in both references. The reads mapping exclusively to the new region were extracted and compared with the reads from the initial mapping against *Y. pestis* and gap-bridging reads were identified. Based on these exclusive mapping reads, the coverage for the *pla−* plasmid was calculated. The coverage of the *pla* region (NC_003132.1, 6,428–8,529) in the regular mapping was used to estimate the number of *pla*+ plasmids. Based on these two values a ratio was calculated to estimate the proportions.

### Human genetic analyses

#### Mapping and aDNA damage patterns

Shotgun sequence data was mapped to the human genome build hg19 using BWA with a reduced mapping stringency parameter “-n 0.01” to account for mismatches in aDNA^54,62^. Duplicates were removed. Authenticity of the aDNA fragments was assessed using mapDamage 2.0^63,64^. After validation of the terminal damage patterns, the first two positions from the 5′ end of the fastq-reads were trimmed.

#### Genotyping

Alleles were drawn at random from a pool of 1,233,013 SNPs (commonly used for population genetic analyses) in a pseudo-haploid manner using a custom script as described previously^65–68^. Datasets had to have at least 10,000 SNPs to be considered for further analysis^68^. Five individuals from Riga (G92, G143b, G161c, G488 and G701) passed this filtering step.

#### Population genetic analyses

The five genotype datasets from Riga were merged with a dataset of previously published 59 West Eurasian populations genotyped on the aforementioned 1,233,013 SNPs using the program mergeit from the EIGENSOFT package^69^. Principal component analysis (PCA) was performed using the software smartpca and the ‘lsqproject’ option^69^. Admixture modelling was done with the software ADMIXTURE on the same populations as used in the PCA. The number of ancestral components k ranged from 4 to 12^70^. Cross-validation was performed for every admixture model and the model with the highest accuracy was determined by the lowest cross-validation error.

#### F3 outgroup statistics

f3 outgroup statistics were run as a part of the Admixtools package in the form of *f3 *(Riga; test; Mbuti) using for test the same populations as used in the PCA and ADMIXTURE analysis^70^.

### Material transfer agreement

The authors confirm that the sampling and the ancient DNA analysis of human bones from the fifteenth–seventeenth century archaeological features unearthed in Riga, Latvia were performed with permission from the institution which headed the excavations and the anthropological analysis (Institute of Latvian History, University of Latvia).

## Supplementary information

Supplementary Information.

## Data Availability

The aligned sequences are available through the European Nucleotide Archive under Accession Number PRJEB36413.
