# Supplementary material for: Yersinia pestis strains from Latvia show depletion of the pla virulence gene at the end of the second plague pandemic
Source: Sci Rep. 2020 Sep 3;10:14628. doi: 10.1038/s41598-020-71530-9 (PMC7471286; doi:10.1038/s41598-020-71530-9)
Supplement: Supplementary file 1 — Supplementary Information. [file 41598_2020_71530_MOESM1_ESM.docx]

**Title:**

*Yersinia pestis* strains from Latvia show depletion of the *pla* virulence gene at the end of the second plague pandemic.

**Authors:**

Julian Susat, Joanna Bonczarowska, Elīna Pētersone-Gordina, Alexander Immel, Almut Nebel, Guntis Gerhards, Ben Krause-Kyora


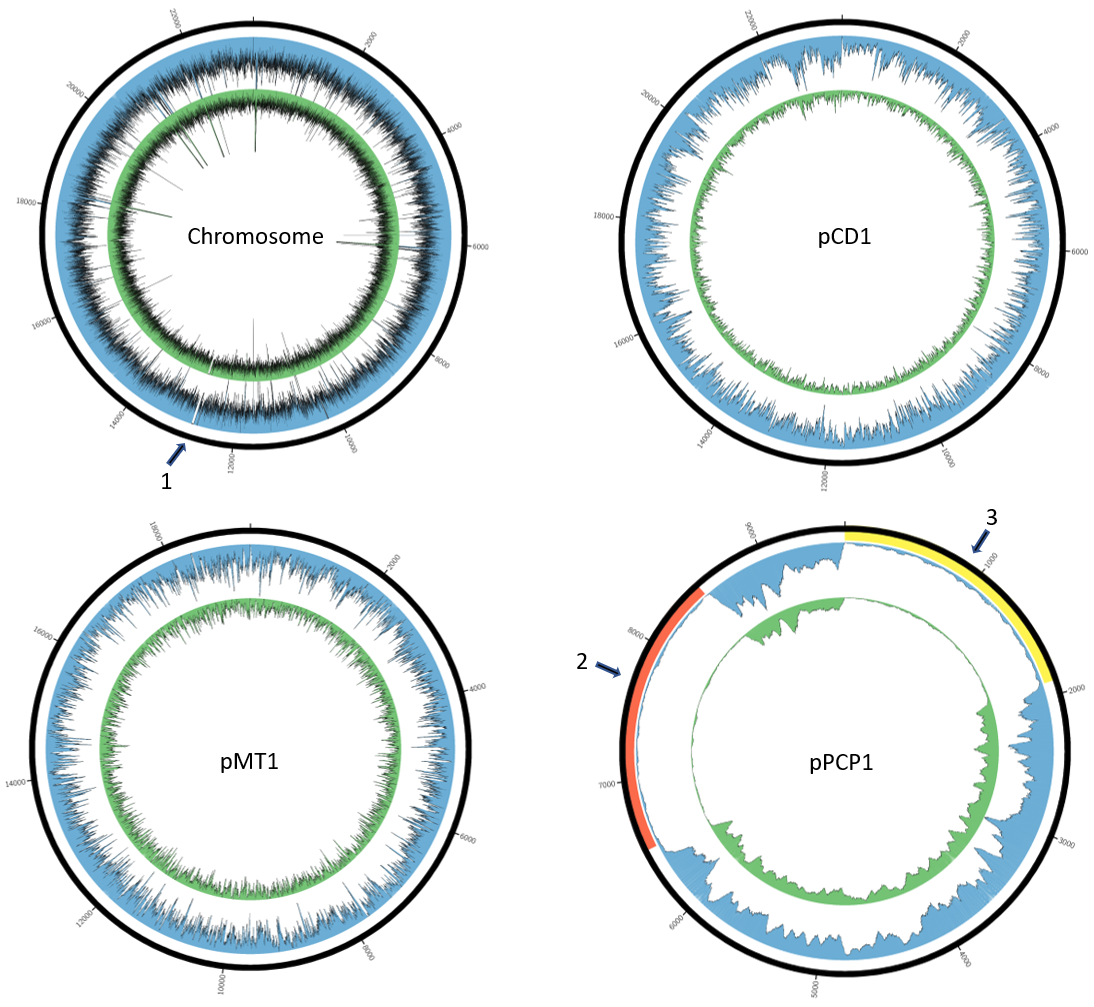


**Figure S1. Average coverage plots for the chromosome and the three plasmids of the two Riga strains G488 (blue) and G701 (green) compared with the CO92 reference.** The average depth of coverage was calculated for 200-bp regions for the chromosome, 3-bp regions for pCD1 and 5-bp regions for pMT1. For a true representation of coverage depth, each plot was given a different threshold for the maximum value. Chromosome 10x, pCD1 40x, pMT1 25x and pPCP1 100x. All figures were generated using Circos. 1: filamentous prophage YpfΦ which is not covered by any sequences in our sample, 2: *pla* gene region 6428-8530 including the *pla* gene (YPPCP1.07), a putative transcriptional regulator (YPPCP1.08c) and a hypothetical protein (YPPCP1.09c), 3: insertion sequence IS100 including a transposase (YPPCP1.01) and an ATP-binding protein (YPPCP1.01). Due to the presence of this region in several parts of the genome, filtering led to a drop in coverage in this region.


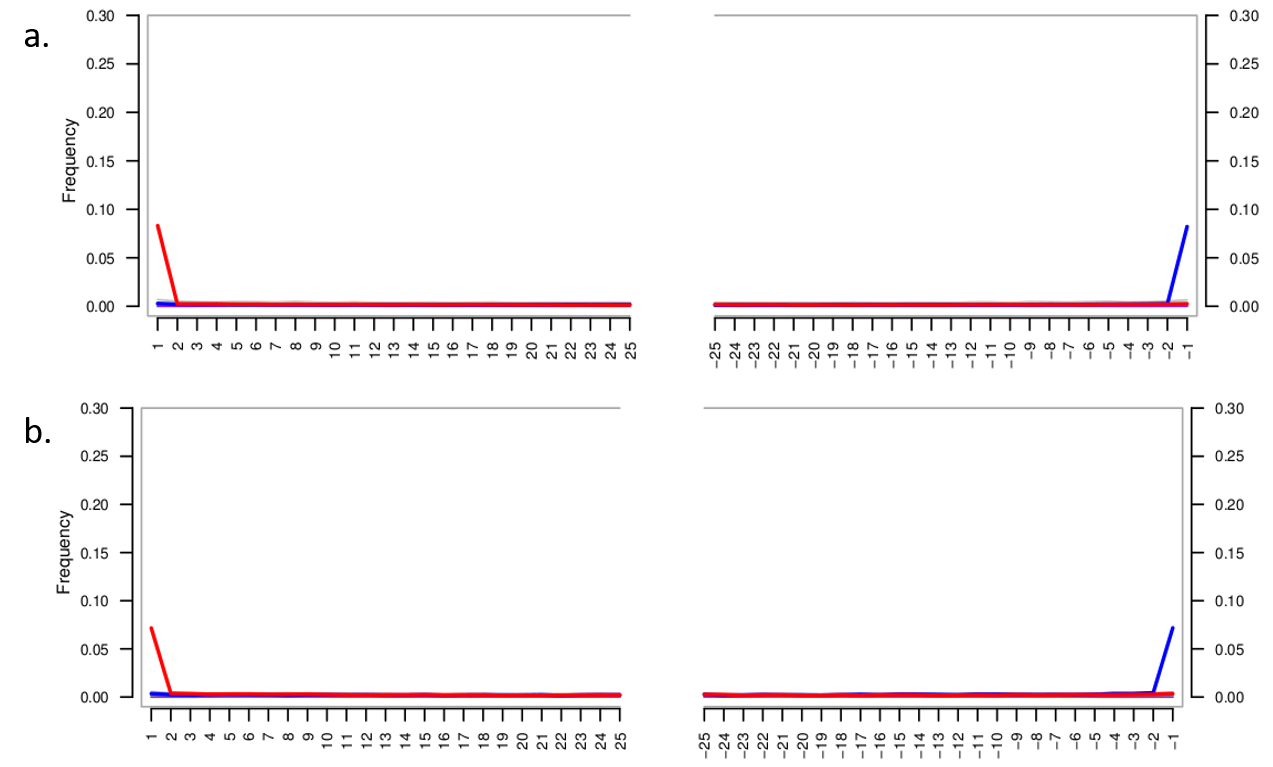


**Figure S2. aDNA damage patterns for G448 (a) and for G701 (b).** Both samples show the expected degradation patterns, increased C > T and G > A substitutions at the 5´ and 3´ends of the reads, after half-UDG library preparation.


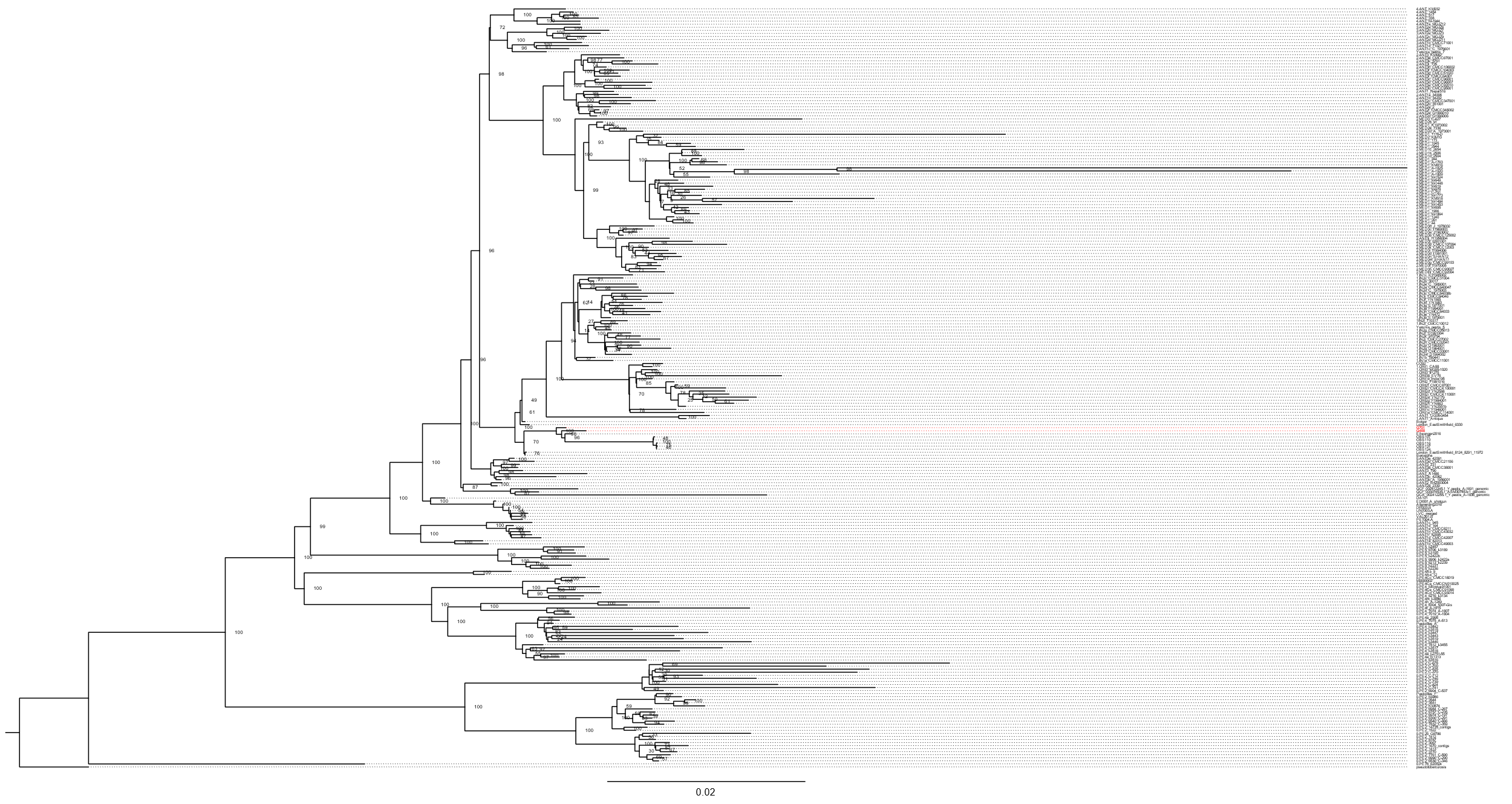


**Figure S3. Maximum-likelihood tree generated with RAxML.** Bootstrap values are shown for 500 replicates.


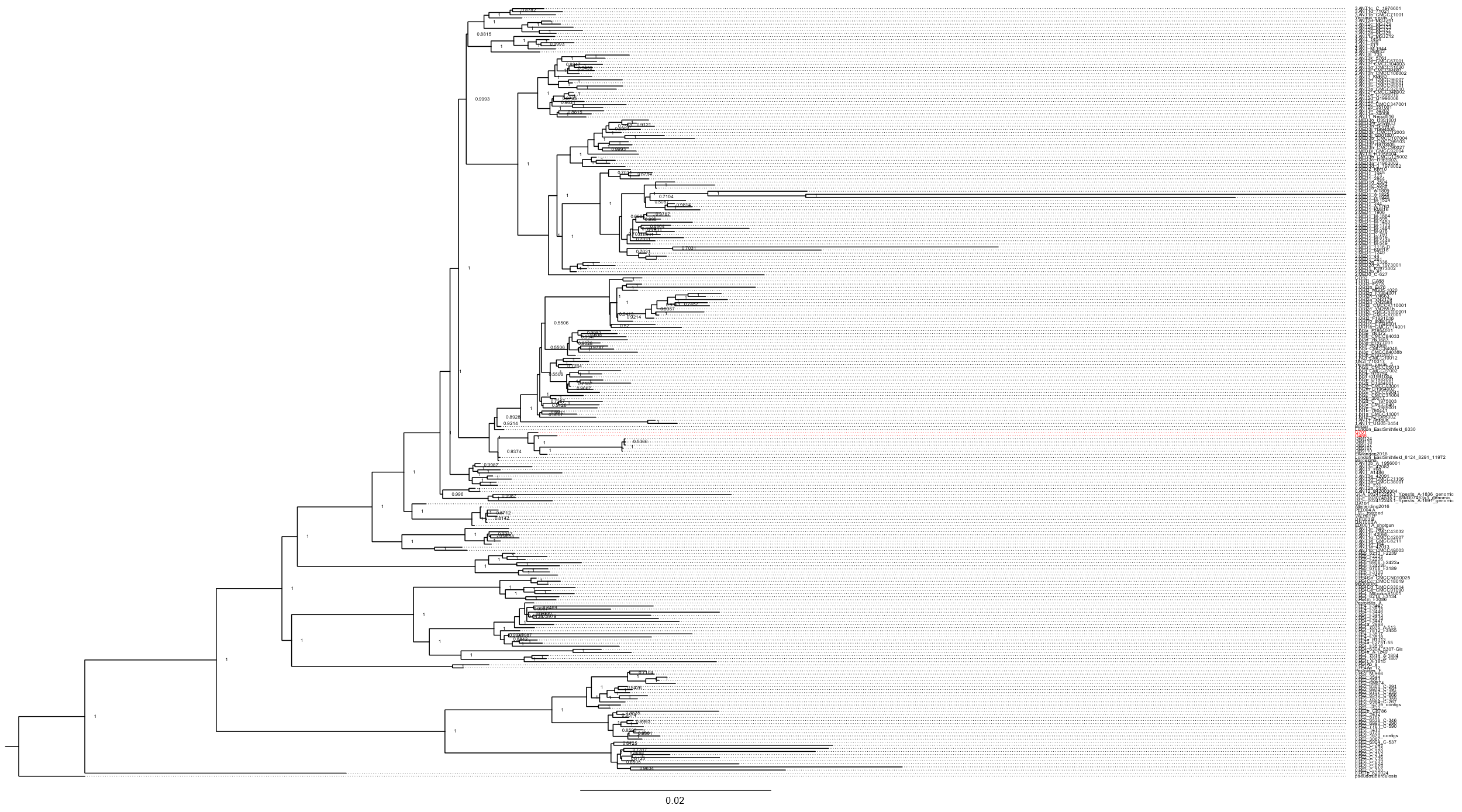


**Figure S4. Bayesian tree generated with MrBayes.** Posterior probability is shown for one million generations.


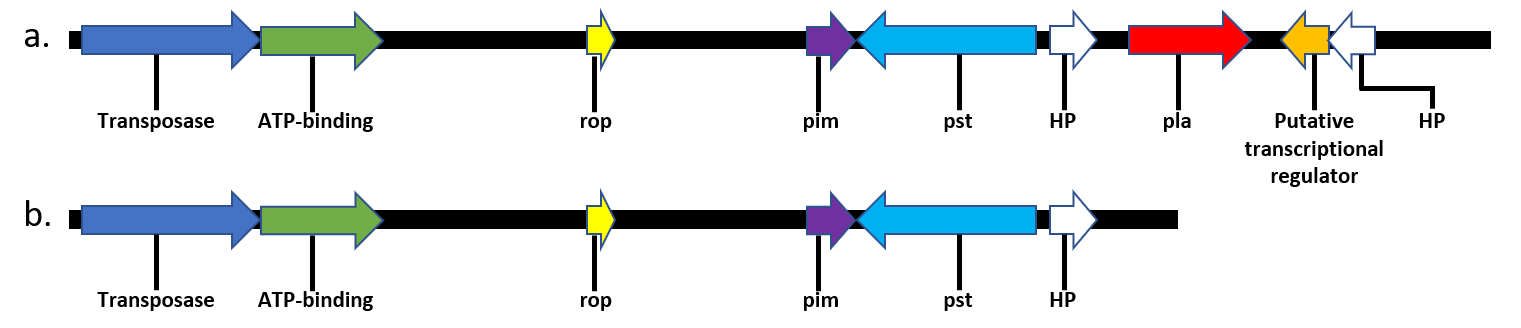


**Figure S5. Schematic linear diagram of the pPCP1 plasmid with the *pla* region (*pla+*, a) and without the *pla* region (*pla-*, b).** *rop*: replication regulation protein, *pim*: transcriptional regulator, *pst*: pesticin, *pla*: plasminogen activator (PLA) protease, *HP*: hypothetical protein.

**
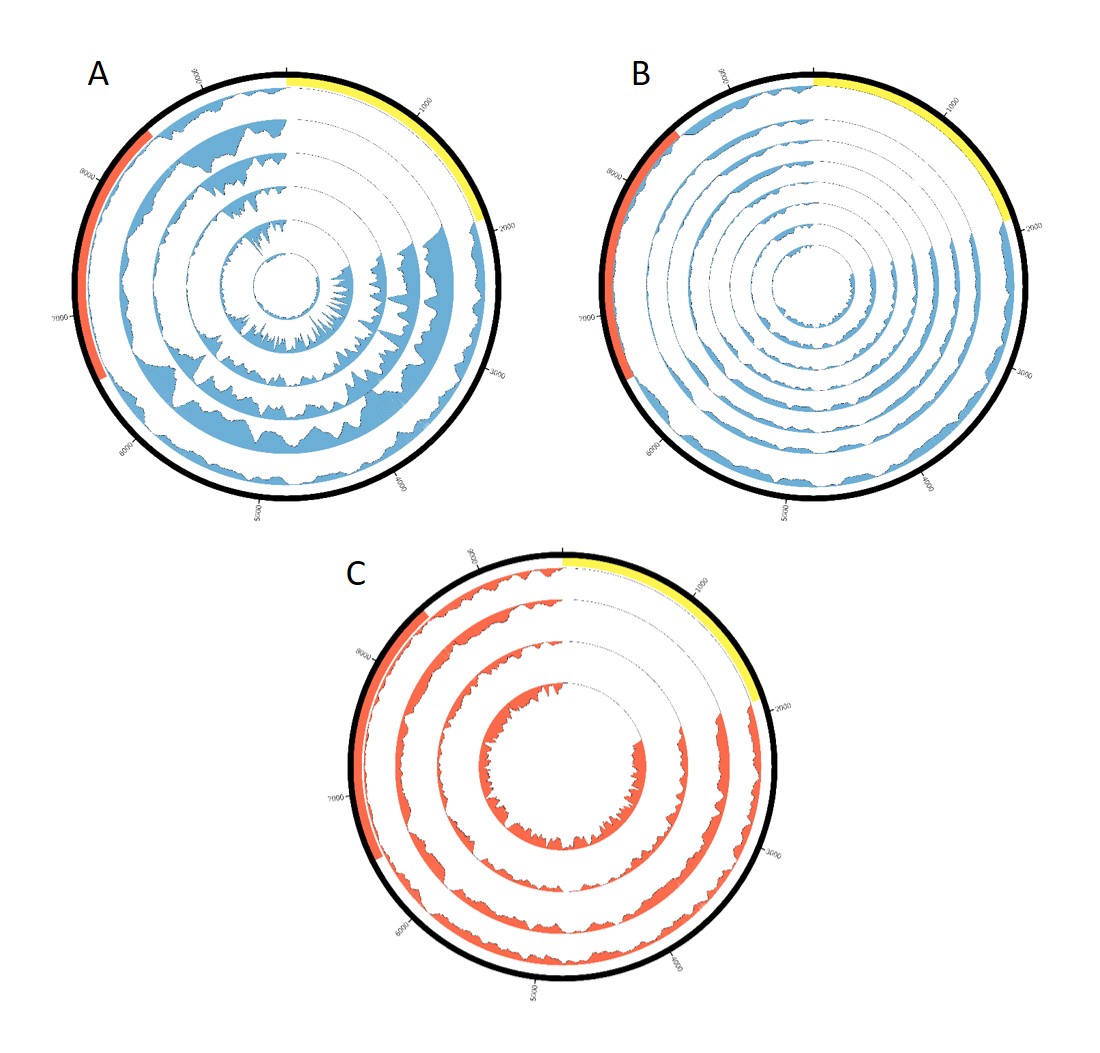
**

**Figure S6. Coverage plots for the pPCP1 plasmid of the 14 strains from the second pandemic that could be analyzed for the depletion of the *pla* region.** A and B depict the coverage of *pla*-depleted strains exhibiting gap-spanning reads (see blue colored strains Fig. 1). C depicts the coverage of the strains where no gap-spanning reads could be found (see red colored strains Fig. 1).

**Figure S7. Principal component analysis (PCA) of the five Riga individuals.** Riga individuals shown in red were projected onto a basemap of 59 modern-day West Eurasian populations. PC1 is shown on the X-axis and PC2 on the Y-axis.

**Figure S8. Detail of the principal component analysis (PCA) of the five Riga individuals.** Riga individuals shown in red were projected onto a basemap of 59 modern-day West Eurasian populations. PC1 is shown on the X-axis and PC2 on the Y-axis.


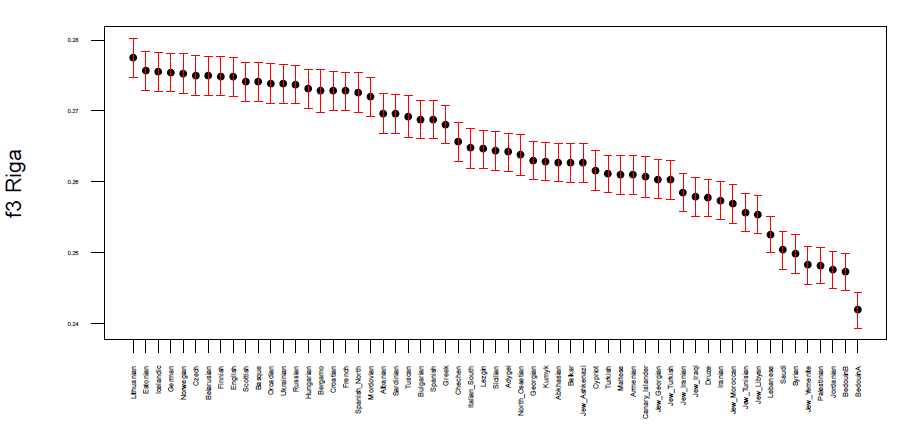


**Figure S9. F3 statistics.** f3 outgroup statistics *f3(Riga; test, Mbuti)* showing the amount of shared genetic drift between the Riga individuals analysed in this study and each of the 59 modern-day West Eurasian populations (*test*) as used in the PCA and admixture analysis.


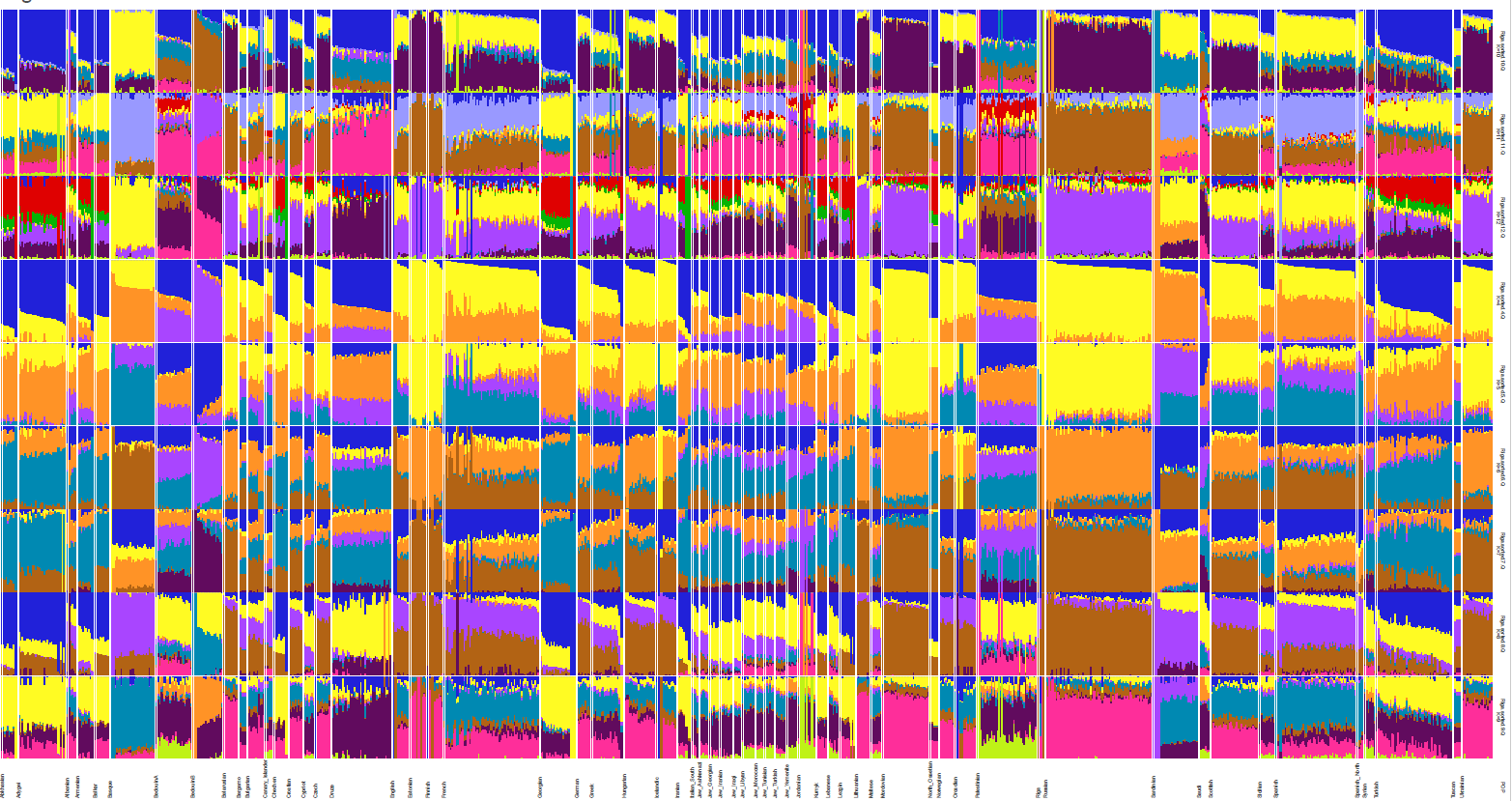


**Figure S10. Admixture plot.** Admixture analysis of the Riga individuals together with 59 modern-day West Eurasian populations (the same as in the PCA) shown for K=4 to K=12 components.

**Table S1. Number of *Y. pestis* reads detected during the initial screening using MALT**

| **Sample** | **Number of reads mapping to NC_003143.1** |
| --- | --- |
| G103 | 8 |
| G488 | 1489 |
| G645 | 199 |
| G701 | 1312 |

**Table S2. *Yersinia* genomes used in the multi fasta reference mapping for authentication of a *Y. pestis-*positive sample**

| **Species name** | **Strain** | **NCBI accession number** |
| --- | --- | --- |
| *Y. pestis* | CO92 | NC_003143.1 |
| *Y. pseudotuberculosis* | IP 32953 | NC_006155.1 |
| *Y. enterocolitica* | Subsp. enterocolitica 8081 | NC_008800.1 |
| *Y. aldovae* | ATCC 35236 | NZ_ACCB01000210.1 |
| *Y. bercovie* | ATCC 43970 | NZ_AALC02000229.1 |
| *Y. frederiksenii* | ATCC 33641 | NZ_AALE02000161.1 |
| *Y. intermedia* | ATCC 29909 | NZ_AALF02000123.1 |
| *Y. kristensenii* | ATCC 33638 | NZ_ACCA01000153.1 |
| *Y. mollaretii* | ATCC 43969 | NZ_AALD02000179.1 |
| *Y. rohdei* | ATCC 43380 | NZ_ACCD01000141.1 |
| *Y. ruckeri* | ATCC 29473 | NZ_ACCC01000174. |

**Table S3. Score calculation for authentication of *Y. pestis-*positive samples**

| **Sample** | **YPS** | **Max(YS)** | **Reads total in sample** | **Score** |
| --- | --- | --- | --- | --- |
| G103 | 16 | 133 | 11662509 | -0.0100 |
| G488 | 1881 | 181 | 8277430 | 0.2053 |
| G645 | 211 | 163 | 11091584 | 0.0043 |
| G701 | 1591 | 113 | 7930905 | 0.1863 |

**Table S4. 228 modern strains used in the SNP-based phylogeny**

| **Strain ID** | **Accession No.** | **Origin** | **Reference** |
| --- | --- | --- | --- |
| 0.ANT1a_42013 | ADPG00000000 | China | Cui et al. 2013 |
| 0.ANT1b_CMCC49003 | ADQX00000000 | China | Cui et al. 2013 |
| 0.ANT1c_945 | ADPV00000000 | China | Cui et al. 2013 |
| 0.ANT1d_164 | ADOW00000000 | China | Cui et al. 2013 |
| 0.ANT1e_CMCC8211 | ADRD00000000 | China | Cui et al. 2013 |
| 0.ANT1f_42095 | ADPJ00000000 | China | Cui et al. 2013 |
| 0.ANT1g_CMCC42007 | ADQV00000000 | China | Cui et al. 2013 |
| 0.ANT1h_CMCC43032 | ADQW00000000 | China | Cui et al. 2013 |
| 0.ANT2_B42003004 | AAYU00000000 | China | Cui et al. 2013 |
| 0.ANT2a_2330 | ADOY00000000 | China | Cui et al. 2013 |
| 0.ANT3_231 | JMUF00000000 | Former Soviet Union | Eroshenko et al. 2017 |
| 0.ANT3_790 | CP006806 | Former Soviet Union | Zhgenti et al. 2015 |
| 0.ANT3_A-1486 | LYMP | Former Soviet Union | Eroshenko et al. 2017 |
| 0.ANT3a_CMCC38001 | ADQU00000000 | China | Cui et al. 2013 |
| 0.ANT3b_A1956001 | ADPX00000000 | China | Cui et al. 2013 |
| 0.ANT3c_42082 | ADPH00000000 | China | Cui et al. 2013 |
| 0.ANT3d_CMCC21106 | ADQP00000000 | China | Cui et al. 2013 |
| 0.ANT3e_42091b | ADPI00000000 | China | Cui et al. 2013 |
| 0.ANT5_A-1691 | LYMQ | Former Soviet Union | Eroshenko et al. 2017 |
| 0.ANT5_A-1836 | LYOL00000000 | Former Soviet Union | Eroshenko et al. 2017 |
| 0.PE2_1412 | CP006783 | Former Soviet Union | Zhgenti et al. 2015 |
| 0.PE2_1413 | CP006762 | Former Soviet Union | Zhgenti et al. 2015 |
| 0.PE2_14735 | AYLS00000000 | Former Soviet Union | Zhgenti et al. 2015 |
| 0.PE2_1522 | CP006758 | Former Soviet Union | Zhgenti et al. 2015 |
| 0.PE2_1670 | CP006806 | Former Soviet Union | Zhgenti et al. 2015 |
| 0.PE2_3067 | CP006754 | Former Soviet Union | Zhgenti et al. 2015 |
| 0.PE2_3544 | LZNH | Former Soviet Union | Kutyrev et al. 2018 |
| 0.PE2_3551 | MBSJ00000000 | Former Soviet Union | Kutyrev et al. 2018 |
| 0.PE2_3770 | CP006751 | Former Soviet Union | Zhgenti et al. 2015 |
| 0.PE2_8787 | CP006748 | Former Soviet Union | Zhgenti et al. 2015 |
| 0.PE2_C-197 | LIYX | Former Soviet Union | Kislichkina et al. 2015 |
| 0.PE2_C-235 | LIYY | Former Soviet Union | Kislichkina et al. 2015 |
| 0.PE2_C-267 | LIYZ | Former Soviet Union | Kislichkina et al. 2015 |
| 0.PE2_C-290 | LIYU | Former Soviet Union | Kislichkina et al. 2015 |
| 0.PE2_C-291 | LIZC | Former Soviet Union | Kislichkina et al. 2015 |
| 0.PE2_C-346 | LIZE | Former Soviet Union | Kislichkina et al. 2015 |
| 0.PE2_C-359 | LIZB | Former Soviet Union | Kislichkina et al. 2015 |
| 0.PE2_C-370 | MIDX00000000 | Former Soviet Union | Kislichkina et al. 2017 |
| 0.PE2_C-535 | MIDY00000000 | Former Soviet Union | Kislichkina et al. 2017 |
| 0.PE2_C-537 | LIYP | Former Soviet Union | Kislichkina et al. 2015 |
| 0.PE2_C-590 | LIYQ | Former Soviet Union | Kislichkina et al. 2015 |
| 0.PE2_C-666 | LIZF | Former Soviet Union | Kislichkina et al. 2015 |
| 0.PE2_C-678 | MIDZ00000000 | Former Soviet Union | Kislichkina et al. 2017 |
| 0.PE2_C-700 | MIEA00000000 | Former Soviet Union | Kislichkina et al. 2017 |
| 0.PE2_C-712 | MTZW00000000 | Former Soviet Union | Kislichkina et al. 2017 |
| 0.PE2_C-739 | MTZX00000000 | Former Soviet Union | Kislichkina et al. 2017 |
| 0.PE2_C-741 | LPTX | Former Soviet Union | Kutyrev et al. 2018 |
| 0.PE2_C-746 | MTZY00000000 | Former Soviet Union | Kislichkina et al. 2017 |
| 0.PE2_C-824 | MTZZ00000000 | Former Soviet Union | Kislichkina et al. 2017 |
| 0.PE2_KM874 | LZTG | Former Soviet Union | Kutyrev et al. 2018 |
| 0.PE2_M-986 | LYMO | Former Soviet Union | Kutyrev et al. 2018 |
| 0.PE2_PEST-F | NC_009381 | Former Soviet Union | Cui et al. 2013 |
| 0.PE2b_G8786 | ADSG00000000 | Former Soviet Union | Cui et al. 2013 |
| 0.PE4_5307-Gis | LIYS | Former Soviet Union | Kislichkina et al. 2015 |
| 0.PE4_A-1804 | LIYW | Former Soviet Union | Kislichkina et al. 2015 |
| 0.PE4_A-1807 | LIYT | Former Soviet Union | Kislichkina et al. 2015 |
| 0.PE4_A-513 | LIZA | Former Soviet Union | Kislichkina et al. 2015 |
| 0.PE4_I-3134 | LIYR | Former Soviet Union | Kislichkina et al. 2015 |
| 0.PE4_I-3442 | NHYH00000000 | Former Soviet Union | Kislichkina et al. 2018a |
| 0.PE4_I-3443 | MIED00000000 | Former Soviet Union | Kislichkina et al. 2018a |
| 0.PE4_I-3446 | NHYI00000000 | Former Soviet Union | Kislichkina et al. 2018a |
| 0.PE4_I-3447 | MIEE00000000 | Former Soviet Union | Kislichkina et al. 2018a |
| 0.PE4_I-3455 | LIYV | Former Soviet Union | Kislichkina et al. 2015 |
| 0.PE4_I-3515 | NHYJ00000000 | Former Soviet Union | Kislichkina et al. 2018a |
| 0.PE4_I-3516 | NHMW00000000 | Former Soviet Union | Kislichkina et al. 2018a |
| 0.PE4_I-3517 | NHMX00000000 | Former Soviet Union | Kislichkina et al. 2018a |
| 0.PE4_I-3518 | NHMY00000000 | Former Soviet Union | Kislichkina et al. 2018a |
| 0.PE4_I-3519 | NHMZ00000000 | Former Soviet Union | Kislichkina et al. 2018a |
| 0.PE4_M0000002 | ADST00000000 | China | Cui et al. 2013 |
| 0.PE4_Microtus91001 | NC_005810 | China | Cui et al. 2013 |
| 0.PE4a_B1313 | LYMS | Former Soviet Union | Kutyrev et al. 2018 |
| 0.PE4a_I-2751-55 | LYCL00000000 | Former Soviet Union | Kutyrev et al. 2018 |
| 0.PE4a_I-2998 | LYMR | Former Soviet Union | Kutyrev et al. 2018 |
| 0.PE4Aa_12 | ADOV00000000 | China | Cui et al. 2013 |
| 0.PE4Ab_9 | ADPT00000000 | China | Cui et al. 2013 |
| 0.PE4Ba_PestoidesA | ACNT00000000 | Former Soviet Union | Cui et al. 2013 |
| 0.PE4Ca_CMCCN010025 | ADRT00000000 | China | Cui et al. 2013 |
| 0.PE4Cc_CMCC18019 | ADQO00000000 | China | Cui et al. 2013 |
| 0.PE4Cd_CMCC93014 | ADRM00000000 | China | Cui et al. 2013 |
| 0.PE4Ce_CMCC91090 | ADRJ00000000 | China | Cui et al. 2013 |
| 0.PE4h_A-1249 | LYMN | Former Soviet Union | Eroshenko et al. 2017 |
| 0.PE4m_I-3086 | LZNY | Mongolia | Kutyrev et al. 2018 |
| 0.PE4t_A-1815 | LPTY | Former Soviet Union | Eroshenko et al. 2017 |
| 0.PE5_I-2231 | PVLX00000000 | Mongolia | Kislichkina et al. 2018b |
| 0.PE5_I-2236 | PVLZ00000000 | Mongolia | Kislichkina et al. 2018b |
| 0.PE5_I-2239 | LIZD | Mongolia | Kislichkina et al. 2015 |
| 0.PE5_I-2422a | LIZG | Mongolia | Kislichkina et al. 2015 |
| 0.PE5_I-2422b | QANK00000000 | Mongolia | Kutyrev et al. 2018 |
| 0.PE5_I-2457 | PVMB00000000 | Mongolia | Kislichkina et al. 2018b |
| 0.PE5_I-3189 | LIYO | Mongolia | Kislichkina et al. 2015 |
| 0.PE5_I-3190 | PVLY00000000 | Mongolia | Kislichkina et al. 2018b |
| 0.PE7b_620024 | ADPM00000000 | China | Cui et al. 2013 |
| 1.ANT1_Antiqua | NC_008150 | Congo | Cui et al. 2013 |
| 1.ANT1_UG05-0454 | AAYR00000000 | Uganda | Cui et al. 2013 |
| 1.IN1a_CMCC11001 | ADQK00000000 | China | Cui et al. 2013 |
| 1.IN1b_780441 | ADPS00000000 | China | Cui et al. 2013 |
| 1.IN1c_K21985002 | ADSS00000000 | China | Cui et al. 2013 |
| 1.IN2a_CMCC640047 | ADRA00000000 | China | Cui et al. 2013 |
| 1.IN2b_30017 | ADPC00000000 | China | Cui et al. 2013 |
| 1.IN2c_CMCC31004 | ADQR00000000 | China | Cui et al. 2013 |
| 1.IN2d_C1975003 | ADPZ00000000 | China | Cui et al. 2013 |
| 1.IN2e_C1989001 | ADQB00000000 | China | Cui et al. 2013 |
| 1.IN2f_710317 | ADPP00000000 | China | Cui et al. 2013 |
| 1.IN2g_CMCC05013 | ADQF00000000 | China | Cui et al. 2013 |
| 1.IN2i_CMCC10012 | ADQG00000000 | China | Cui et al. 2013 |
| 1.IN2j_CMCC27002 | ADQQ00000000 | China | Cui et al. 2013 |
| 1.IN2k_970754 | ADPW00000000 | China | Cui et al. 2013 |
| 1.IN2l_D1991004 | ADRX00000000 | China | Cui et al. 2013 |
| 1.IN2m_D1964002b | ADRV00000000 | China | Cui et al. 2013 |
| 1.IN2n_CMCC02041 | ADQC00000000 | China | Cui et al. 2013 |
| 1.IN2o_CMCC03001 | ADQD00000000 | China | Cui et al. 2013 |
| 1.IN2p_D1982001 | ADRW00000000 | China | Cui et al. 2013 |
| 1.IN2q_D1964001 | ADRU00000000 | China | Cui et al. 2013 |
| 1.IN3a_F1954001 | ADSC00000000 | China | Cui et al. 2013 |
| 1.IN3b_E1979001 | AAYV00000000 | China | Cui et al. 2013 |
| 1.IN3c_CMCC84038b | ADRF00000000 | China | Cui et al. 2013 |
| 1.IN3d_YN1683 | ADTD00000000 | China | Cui et al. 2013 |
| 1.IN3e_YN472 | ADTH00000000 | China | Cui et al. 2013 |
| 1.IN3f_YN1065 | ADTC00000000 | China | Cui et al. 2013 |
| 1.IN3g_E1977001 | ADRY00000000 | China | Cui et al. 2013 |
| 1.IN3h_CMCC84033 | ADRE00000000 | China | Cui et al. 2013 |
| 1.IN3i_CMCC84046 | ADRG00000000 | China | Cui et al. 2013 |
| 1.ORI1_CA88 | ABCD00000000 | USA | Cui et al. 2013 |
| 1.ORI1_CO92 | NC_003143 | USA | Cui et al. 2013 |
| 1.ORI1a_CMCC114001 | ADQL00000000 | China | Cui et al. 2013 |
| 1.ORI1b_India195 | ACNR00000000 | India | Cui et al. 2013 |
| 1.ORI1c_F1946001 | ADSB00000000 | China | Cui et al. 2013 |
| 1.ORI2_F1991016 | ABAT00000000 | China | Cui et al. 2013 |
| 1.ORI2a_YN2179 | ADTE00000000 | Myanmar | Cui et al. 2013 |
| 1.ORI2c_YN2551b | ADTF00000000 | China | Cui et al. 2013 |
| 1.ORI2d_YN2588 | ADTG00000000 | China | Cui et al. 2013 |
| 1.ORI2f_CMCC87001 | ADRH00000000 | China | Cui et al. 2013 |
| 1.ORI2g_F1984001 | ADSD00000000 | China | Cui et al. 2013 |
| 1.ORI2h_YN663 | ADTI00000000 | China | Cui et al. 2013 |
| 1.ORI2i_CMCCK100001a | ADRR00000000 | China | Cui et al. 2013 |
| 1.ORI2i_CMCCK110001b | ADRS00000000 | China | Cui et al. 2013 |
| 1.ORI3_IP275 | AAOS00000000 | Madagascar | Cui et al. 2013 |
| 1.ORI3_MG05-1020 | AAYS00000000 | Madagascar | Cui et al. 2013 |
| 1.ORI3a_EV76 | ADSA00000000 | Madagascar | Cui et al. 2013 |
| 2.ANT1_Nepal516 | ACNQ00000000 | Nepal | Cui et al. 2013 |
| 2.ANT1a_34008 | ADPD00000000 | China | Cui et al. 2013 |
| 2.ANT1b_34202 | ADPE00000000 | China | Cui et al. 2013 |
| 2.ANT2a_2 | ADOX00000000 | China | Cui et al. 2013 |
| 2.ANT2b_351001 | ADPF00000000 | China | Cui et al. 2013 |
| 2.ANT2c_CMCC347001 | ADQS00000000 | China | Cui et al. 2013 |
| 2.ANT2d_G1996006 | ADSE00000000 | China | Cui et al. 2013 |
| 2.ANT2e_G1996010 | ADSF00000000 | China | Cui et al. 2013 |
| 2.ANT2f_CMCC348002 | ADQT00000000 | China | Cui et al. 2013 |
| 2.ANT3_KM682 | LPVG | Former Soviet Union | Kutyrev et al. 2018 |
| 2.ANT3a_CMCC92010 | ADRL00000000 | China | Cui et al. 2013 |
| 2.ANT3b_CMCC95001 | ADRN00000000 | China | Cui et al. 2013 |
| 2.ANT3c_CMCC96001 | ADRO00000000 | China | Cui et al. 2013 |
| 2.ANT3d_CMCC96007 | ADRP00000000 | China | Cui et al. 2013 |
| 2.ANT3e_CMCC67001 | ADRB00000000 | China | Cui et al. 2013 |
| 2.ANT3f_CMCC104003 | ADQH00000000 | China | Cui et al. 2013 |
| 2.ANT3g_CMCC51020 | ADQY00000000 | China | Cui et al. 2013 |
| 2.ANT3h_CMCC106002 | ADQI00000000 | China | Cui et al. 2013 |
| 2.ANT3i_CMCC64001 | ADQZ00000000 | China | Cui et al. 2013 |
| 2.ANT3j_H1959004 | ADSI00000000 | China | Cui et al. 2013 |
| 2.ANT3k_5761 | ADPL00000000 | Russia | Cui et al. 2013 |
| 2.ANT3l_735 | ADPR00000000 | Russia | Cui et al. 2013 |
| 2.MED0_C-627 | MBSI00000000 | Former Soviet Union | Kutyrev et al. 2018 |
| 2.MED1_1045 | CP006794 | Former Soviet Union | Zhgenti et al. 2015 |
| 2.MED1_1116-D | LPXS | Former Soviet Union | Kutyrev et al. 2018 |
| 2.MED1_1240 | LZNI | Former Soviet Union | Kutyrev et al. 2018 |
| 2.MED1_139 | QAPA00000000 | Former Soviet Union | Kutyrev et al. 2018 |
| 2.MED1_173 | LQAZ | Former Soviet Union | Kutyrev et al. 2018 |
| 2.MED1_1906 | LYOM | Former Soviet Union | Kutyrev et al. 2018 |
| 2.MED1_244 | LZND | Former Soviet Union | Kutyrev et al. 2018 |
| 2.MED1_261 | LZNG | Former Soviet Union | Kutyrev et al. 2018 |
| 2.MED1_2944 | CP006792 | Former Soviet Union | Zhgenti et al. 2015 |
| 2.MED1_44 | LZNF | Former Soviet Union | Kutyrev et al. 2018 |
| 2.MED1_A-1763 | LQAW | Former Soviet Union | Kutyrev et al. 2018 |
| 2.MED1_A-1809 | LYMF | Former Soviet Union | Eroshenko et al. 2017 |
| 2.MED1_A-1825 | LYCM | Former Soviet Union | Kutyrev et al. 2018 |
| 2.MED1_A-1920 | LYCO | Former Soviet Union | Kutyrev et al. 2018 |
| 2.MED1_C-791 | LQAU | Former Soviet Union | Kutyrev et al. 2018 |
| 2.MED1_K1973002 | AAYT00000000 | China | Cui et al. 2013 |
| 2.MED1_KIM10 | NC_004088 | Iran | Cui et al. 2013 |
| 2.MED1_KM816 | LPXU | Former Soviet Union | Kutyrev et al. 2018 |
| 2.MED1_KM918 | LPQY | Former Soviet Union | Kutyrev et al. 2018 |
| 2.MED1_M-1448 | LYCN | Former Soviet Union | Kutyrev et al. 2018 |
| 2.MED1_M-1453 | LQAY | Former Soviet Union | Kutyrev et al. 2018 |
| 2.MED1_M-1484 | LQAV | Former Soviet Union | Kutyrev et al. 2018 |
| 2.MED1_M-1524 | LYCP | Former Soviet Union | Kutyrev et al. 2018 |
| 2.MED1_M-1773 | LYMG | Former Soviet Union | Kutyrev et al. 2018 |
| 2.MED1_M-1864 | LOHR | Former Soviet Union | Kutyrev et al. 2018 |
| 2.MED1_M-519 | LQAX | Former Soviet Union | Kutyrev et al. 2018 |
| 2.MED1_M-549 | LQBA | Former Soviet Union | Kutyrev et al. 2018 |
| 2.MED1_M-595 | LYOH | Former Soviet Union | Kutyrev et al. 2018 |
| 2.MED1_M-978 | LPXT | Former Soviet Union | Kutyrev et al. 2018 |
| 2.MED1b_2506 | ADPA00000000 | China | Cui et al. 2013 |
| 2.MED1c_2654 | ADPB00000000 | China | Cui et al. 2013 |
| 2.MED1d_2504 | ADOZ00000000 | China | Cui et al. 2013 |
| 2.MED2b_91 | ADPU00000000 | China | Cui et al. 2013 |
| 2.MED2c_K11973002 | AAYT00000000 | China | Cui et al. 2013 |
| 2.MED2d_A1973001 | ADPY00000000 | China | Cui et al. 2013 |
| 2.MED2e_7338 | ADPQ00000000 | China | Cui et al. 2013 |
| 2.MED3a_J1963002 | ADSP00000000 | China | Cui et al. 2013 |
| 2.MED3b_CMCC125002b | ADQN00000000 | China | Cui et al. 2013 |
| 2.MED3c_I1969003 | ADSK00000000 | China | Cui et al. 2013 |
| 2.MED3d_J1978002 | ADSQ00000000 | China | Cui et al. 2013 |
| 2.MED3f_I1970005 | ADSL00000000 | China | Cui et al. 2013 |
| 2.MED3g_CMCC99103 | ADRQ00000000 | China | Cui et al. 2013 |
| 2.MED3h_CMCC90027 | ADRI00000000 | China | Cui et al. 2013 |
| 2.MED3i_CMCC92004 | ADRK00000000 | China | Cui et al. 2013 |
| 2.MED3j_I2001001 | ADSO00000000 | China | Cui et al. 2013 |
| 2.MED3k_CMCC12003 | ADQM00000000 | China | Cui et al. 2013 |
| 2.MED3l_I1994006 | ADSN00000000 | China | Cui et al. 2013 |
| 2.MED3m_SHAN11 | ADTA00000000 | China | Cui et al. 2013 |
| 2.MED3n_SHAN12 | ADTB00000000 | China | Cui et al. 2013 |
| 2.MED3o_I1991001 | ADSM00000000 | China | Cui et al. 2013 |
| 2.MED3p_CMCC107004 | ADQJ00000000 | China | Cui et al. 2013 |
| 3.ANT1a_7b | ADPN00000000 | China | Cui et al. 2013 |
| 3.ANT1b_CMCC71001 | ADRC00000000 | China | Cui et al. 2013 |
| 3.ANT1c_C1976001 | ADQA00000000 | China | Cui et al. 2013 |
| 3.ANT1d_71021 | ADPO00000000 | China | Cui et al. 2013 |
| 3.ANT2a_MGJZ6 | ADSX00000000 | Mongolia | Cui et al. 2013 |
| 3.ANT2b_MGJZ7 | ADSY00000000 | Mongolia | Cui et al. 2013 |
| 3.ANT2c_MGJZ9 | ADSZ00000000 | Mongolia | Cui et al. 2013 |
| 3.ANT2d_MGJZ11 | ADSU00000000 | Mongolia | Cui et al. 2013 |
| 3.ANT2e_MGJZ3 | ADSW00000000 | Mongolia | Cui et al. 2013 |
| 4.ANT_1454 | LZNC | Former Soviet Union | Kutyrev et al. 2018 |
| 4.ANT_338 | LZNX | Former Soviet Union | Kutyrev et al. 2018 |
| 4.ANT_517 | LYMH | Former Soviet Union | Kutyrev et al. 2018 |
| 4.ANT_KM932 | LZNE | Former Soviet Union | Kutyrev et al. 2018 |
| 4.ANT_M-1944 | LYOK | Former Soviet Union | Kutyrev et al. 2018 |
| 4.ANT1a_MGJZ12 | ADSV00000000 | Mongolia | Cui et al. 2013 |

**Table S5. 36 ancient strains used in the SNP-based phylogeny**

| **Strain ID** | **Accession No.** | **Origin** | **Reference** |
| --- | --- | --- | --- |
| DA101 | PRJEB25891 | Kyrgyzstan | Damgaard et al. 2018 |
| Altenerding | PRJEB14851 | Germany | Feldman et al. 2016 |
| London_EastSmithfield_8124_8291_11972 | SRR341961,SRR341962,SRR341963 | United Kingdom | Bos et al. 2011 |
| London_EastSmithfield_6330 | SAMN00715799 | United Kingdom | Bos et al. 2011 |
| Ellwangen | PRJEB13664 | Germany | Spyrou et al. 2016 |
| Bolgar | PRJEB13664 | Russia | Spyrou et al. 2016 |
| Barcelona | PRJEB13664 | Spain | Spyrou et al. 2016 |
| OBS107 | PRJEB12163 | France | Bos et al. 2016 |
| OBS110 | PRJEB12163 | France | Bos et al. 2016 |
| OBS116 | PRJEB12163 | France | Bos et al. 2016 |
| OBS124 | PRJEB12163 | France | Bos et al. 2016 |
| OBS137 | PRJEB12163 | France | Bos et al. 2016 |
| EDI001.A | SAMEA5661363 | United Kingdom | Keller et al. 2019 |
| LVC_merged | SAMEA5661382, SAMEA5661378, SAMEA5661380,  SAMEA5661379 | France | Keller et al. 2019 |
| DIT003.B | SAMEA5661360 | Germany | Keller et al. 2019 |
| PET004.A | SAMEA5661385 | Germany | Keller et al. 2019 |
| UNT003.A | SAMEA5661389 | Germany | Keller et al. 2019 |
| VAL001.B | SAMEA5661384 | Spain | Keller et al. 2019 |
| LAI009 | PRJEB29990 | Russia | Spyrou et al. 2019 |
| NAB003 | PRJEB29990 | Germany | Spyrou et al. 2019 |
| MAN008 | PRJEB29990 | Germany | Spyrou et al. 2019 |
| STA001 | PRJEB29990 | Germany | Spyrou et al. 2019 |
| NMS002 | PRJEB29990 | Cambridge | Spyrou et al. 2019 |
| BRA001 | PRJEB29990 | United Kingdom | Spyrou et al. 2019 |
| BED024 | PRJEB29990 | United Kingdom | Spyrou et al. 2019 |
| BED028 | PRJEB29990 | United Kingdom | Spyrou et al. 2019 |
| BED030 | PRJEB29990 | United Kingdom | Spyrou et al. 2019 |
| BED034 | PRJEB29990 | United Kingdom | Spyrou et al. 2019 |
| STN002 | PRJEB29990 | Sweden | Spyrou et al. 2019 |
| STN007 | PRJEB29990 | Sweden | Spyrou et al. 2019 |
| STN008 | PRJEB29990 | Sweden | Spyrou et al. 2019 |
| STN013 | PRJEB29990 | Sweden | Spyrou et al. 2019 |
| STN014 | PRJEB29990 | Sweden | Spyrou et al. 2019 |
| STN019 | PRJEB29990 | Sweden | Spyrou et al. 2019 |
| STN020 | PRJEB29990 | Sweden | Spyrou et al. 2019 |
| STN021 | PRJEB29990 | Sweden | Spyrou et al. 2019 |

**Table S6. SNP description of diagnostic branch-1 positions in the newly sequenced *Y. pestis* genomes G701 and G488**

| **SNP name** | **Chromosome position in CO92** | **CO92** | **G701** | **G488** | **Gene** |
| --- | --- | --- | --- | --- | --- |
| p1 | 189227 | C | C | C | pabA |
| p2 | 1871476 | G | G | G | NC |
| p3 | 699494 | A | G | G | alt(rpoD) |
| p4 | 2262577 | T | G | G | YPO1990 |
| p5 | 4301295 | G | G | G | recQ |
| p6 | 3806677 | C | T | T | b0125(hpt) |
| p7 | 3643387 | G | G | G | YP03271 |

**Table S7. References used in the competitive alignment for the *pla* gene**

| **Reference** | **Organism** | **Type** |
| --- | --- | --- |
| NC_003143.1 | Y. *pestis* | chromosome |
| NC_003134.1 | Y. *pestis* | plasmid |
| NC_003132.1 | Y. *pestis* | plasmid |
| NC_003131.1 | Y. *pestis* | plasmid |
| LK931337.1 | *Citrobacter koseri* | scaffold |
| LM995843.1 | *Escherichia coli* | scaffold |

**Table S8. References used in the analysis of the putative gap-bridging reads**

| NC_003143.1 |
| --- |
| NC_003134.1 |
| NC_003132.1 |
| pla+_NC_003131.1 |
| pla-_NC_003132.1 |

**Table S9. Basic coverage information of the *pla+* and *pla-* plasmids and calculated ratio**

| **Strain** | **Estimated coverage of *pla+*** | **Estimated coverage *pla-*** | **Ratio *pla+/pla-*** |
| --- | --- | --- | --- |
| G701 | 3x | 32x | 1:11 |
| G488 | 7x | 83x | 1:11 |

**Table S10. Basic coverage information of the *pla+* and *pla-* plasmid in the 14 re-analyzed strains^*^ and calculated ratio**

| **Strain** | **Estimated coverage of *pla+*** | **Estimated coverage of *pla-*** | **Ratio *pla+/pla-*** |
| --- | --- | --- | --- |
| BED024 | 9 | 36 | 1:4 |
| BED028 | 22 | 67 | 1:3 |
| BED030 | 46 | 130 | 1:3 |
| BED034 | 12 | 37 | 1:3 |
| BRA001 | 19 | 26 | 1:1 |
| NMS002 | 10 | 31 | 1:3 |
| STN002 | 8 | 17 | 1:2 |
| STN007 | 10 | 40 | 1:4 |
| STN008 | 5 | 18 | 1:4 |
| STN013 | 6 | 19 | 1:3 |
| STN014 | 28 | 46 | 1:2 |
| STN019 | 10 | 28 | 1:3 |
| STN020 | 16 | 41 | 1:3 |
| STN021 | 12 | 30 | 1:3 |

^*^Data for these strains were generated using a *Y. pestis* capture^18^
